# Silicon Carbide-Coated Ceramic Membrane Bioreactor for Sustainable Water Purification

**DOI:** 10.3390/membranes9040047

**Published:** 2019-04-02

**Authors:** Sowmya Surappanahalli Rajanna, Gattumane Motappa Madhu, Chengala Damodara Madhusoodana, Adithya Govindarajan

**Affiliations:** 1 Department of Chemical Engineering, Ramaiah Institute of Technology, Bengaluru, Karnataka 560054, India; adg0707@gmail.com; 2 BHEL, Prof. CNR Rao Circle, IISc Post, Malleswaram, Bengaluru, Karnataka 560012, India; cdm@bhel.in

**Keywords:** ceramic membrane bioreactor, silicon carbide coatings, water purification, permeate flux, fouling, standard pore blocking model, complete pore plugging model

## Abstract

In the present study, a submerged ceramic membrane bioreactor was used to effectively treat industrial wastewater. The outcome of membrane coatings on the efficacy of the membrane was investigated using a silicon carbide (SiC) coating. The flux data obtained from the study were fitted into two mathematical models, namely, the standard pore blocking model (SPBM) and the complete pore plugging model (CPPM) in order to determine the fouling mechanism. It was observed that the SPBM fit with a minimum coefficient of regression of 0.95, suggesting that particles retained on the pore walls were smaller than the average size of membrane pores. An increase in dissolved oxygen (DO) of up to 225% was noted. The significant improvement of the water quality in terms of DO, chemical oxygen demand (COD) and turbidity of coated membrane emphasizes the fact that the membrane coating increases the efficacy of water treatment in membrane bioreactors.

## 1. Introduction

Membrane separation is an efficient filtration process which has gained prevalence in wastewater treatment and recycling owing to its stability and outstanding effluent quality [1,2,3,4,5,6,7,8,9,10]. Between the two configurations, namely, the side stream membrane bioreactor and the submerged membrane bioreactor, the latter stands out as the more effective system owing to its compact size, lower energy consumption, efficient separation of solid-liquid mixture, and relatively lower sludge production [11,12,13,14,15,16,17]. Presently, two different kinds of membranes are used in membrane bio reactor (MBR) systems: ceramic membranes and polymeric membranes [18]. Polymeric membranes are widely used in domestic wastewater purification, whereas ceramic membranes are mainly used for industrial wastewater treatment and are also used for domestic sludge treatment [19,20,21,22,23].

Ceramic membranes possess good strength, good resistance to abrasion of chemicals and biological degradation, eminent permeate flux, high life span, tolerance to high suction pressure and ease for backwashing, thus making the membrane cost effective [24,25,26,27,28]. Ideal membranes should possess properties such as higher water flux, good solute rejection (eminent water purity), chemical, mechanical and thermal steadiness, optimum operating conditions, minimum easy pre-treatment (back flushing and chemical treatment), the ability to be scaled up into large scale membranes and modules, and low capital cost [29,30,31]. Thus, MBR systems using ceramic membranes represent one of the best technologies, owing to the superior quality of the effluent, lesser footprint, excellent organic loading, and lessened production of sludge [32,33,34,35,36].

Nonetheless, the principal drawback of MBR systems is the fouling mechanism encountered in the membrane’s operation [37,38]. Fouling is associated with the accumulation of foulants on the membrane surface, thus reducing the permeate flux [39,40,41,42,43,44]. The reported literature is aimed at pressurized filtration such as dead-end filtration and cross-flow filtration membranes. No significant studies have been reported on vacuum filtration along with aeration. Filtration along with aeration will reduce the fouling rate and improve the filtration rate (flux). Power consumption for vacuum filtration is lower compared to pressure filtration. The primary objectives of the present study are the following: (i) to treat wastewater by subjecting it to microfiltration assisted by vacuum filtration and obtain very good quality with respect to the effluent water; and (ii) to study the effect of SiC coating on the ceramic membrane. For this study, efforts were made to use a fully functional SiC-coated submerged ceramic membrane bioreactor equipped with vacuum microfiltration for effective wastewater purification. SiC was chosen for coating owing to its properties, which include high strength, high hardness, low thermal expansion, excellent thermal shock resistance and chemical inertness. The MBR’s efficacy was studied on the basis of permeate flux, as well as the feed and effluent quality. The pore size of the membrane was analyzed using the scanning electron microscopy (SEM) technique, the studies on permeate flux were carried out using uncoated and the SiC-coated membranes, and the quality of permeate obtained was compared with that of the others. The main parameters, such as chemical oxygen demand (COD), turbidity and dissolved oxygen (DO), were analyzed. The fouling mechanism was investigated by fitting the data obtained by filtration into mathematical models, namely, the standard pore blocking (SPBM) and complete pore plugging (CPPM) mathematical models. Finally, the membranes were subjected to regeneration to revive the permeate flux. In the field of Membrane Technology; the scope of research on coating is wide. The main focus of the present work was to study the effect of coating on ceramic membranes using silicon carbide and analyze the fouling mechanism using two mathematical models.

## 2. Materials and Methods

### 2.1. Materials

Silicon carbide powder, polyvinyl butyral (PVB), ethanol, SB (Super Bond) powder, ethylene glycol, acetone, tap water, urea, meat extract, yeast extract, calcium chloride dihydrate (CaCl_2_·2H_2_O), anhydrous dipotassium hydrogen phosphate (K_2_HPO_4_), magnesium sulphate heptahydrate (MgSO_4_·7H_2_O), and sodium chloride (NaCl) were used for the synthesis of the fermentation feed. The SiC membrane was procured from Bharat Heavy Electricals Limited (Bangalore, India).

The activated sludge was procured from Hikal Ltd. (Bangalore, India) and mixed with the feed. Standard potassium dichromate (K_2_Cr_2_O_7_), concentrated sulfuric acid (H_2_SO_4_), mercuric sulphate (HgSO_4_), ferrous ammonium sulphate hexahydrate (Fe (NH_4_)_2_(SO_4_)_2_·6H_2_O), silversulphate (AgSO_4_), ferrous ammonium sulphate (FAS), and ferroin indicator were used for the COD test. All the chemicals were obtained from Merck (St. Louis, MI, USA), Nice Chemicals, (Kochi, India) and Thomas Baker (Mumbai, India), and were of AR Grade.

Merck, Sigma-Aldrich (St. Louis, MI, USA): yeast extract, anhydrous dipotassium hydrogen phosphate (K_2_HPO_4_), silicon carbide powder, polyvinyl butyral (PVB), acetone, concentrated sulfuric acid (H_2_SO_4_), silver sulphate (AgSO_4_).

Nice Chemicals (Kochi, India): meat extract, sodium chloride, magnesium sulphate heptahydrate (MgSO_4_·7H_2_O), standard potassium dichromate (K_2_Cr_2_O_7_), mercuric sulphate (HgSO_4_), ferrous ammonium sulphate hexahydrate (Fe (NH_4_)_2_(SO_4_)_2_·6H_2_O), ferrous ammonium sulphate (FAS).

Thomas Baker (Mumbai, India): urea, calcium chloride dihydrate (CaCl_2_·2H_2_O), ethylene glycol, ferroin indicator.

### 2.2. Silicon Carbide (SiC) Membrane Coating

The membrane was coated using SiC slurry which was prepared by suspending SiC powder in ethanol along with a polymer binder such as polyvinyl butyral (PVB). The mixture was stirred for half an hour and ethylene glycol (dispersant) was introduced dropwise into the slurry, followed by rigorous stirring for three hours. The membranes were coated using a simple dip-coating technique [45]. After coating, the membranes were dried in a hot air oven at 100 °C for two hours. The membranes are then fired in a furnace at 1250 °C for two hours. Temperature scaling of 10 °C per minute for heating as well as cooling was maintained to ensure removal of the polymeric binder and to strengthen the SiC tubes.

### 2.3. Artificial Fermentation Feed

The fermentation feed was prepared synthetically in the laboratory and consisted of the following components dissolved in each liter of tap water:

160 mg of yeast extract, 110 mg of meat extract; 30 mg of urea, 28 mg of anhydrous dipotassium hydrogen phosphate (K_2_HPO_4_), 7 mg of sodium chloride (NaCl), 4 mg of calcium chloride dihydrate (CaCl_2_·2H_2_O) and 2 mg of magnesium sulphate heptahydrate (MgSO_4_·7H_2_O).

### 2.4. Experimental Procedure

Microfiltration using submerged tubular ceramic membranes was carried out on a batch scale. Each cycle was operated for 24 h. The uncoated membranes were 2 mm thick, 18 cm long, 12.5 mm in diameter with an external surface area of 8.2797 × 10^−3^ m^2^ per tube and pore size of 1.5 microns. Four tubular coated membranes were used which had a thickness of 2 mm + 200 μm coating, a length of 18 cm, a pore size of 0.5 microns, and a diameter of 12.5 mm, with an external surface area of 8.2797 × 10^−3^ m^2^ per tube. The membrane was made up of coarse SiC, and the coating was made using fine SiC. An acrylic tank procured from New Tech Engineers (Bangalore, India) was filled with 10 L of synthetic wastewater. The four tubular ceramic membranes were submerged inside the fermentation medium. Activated sludge was added to the tank to provide the biological environment so that the conventional activated sludge process could be integrated with the modern membrane separation process. A concentration of 5 mL/L of suspended solid was used in the MBR system. Mechanical stirring was provided to homogenize the bioreactor feed. Aeration was provided using an air blower procured from Elico Ltd. (Hyderabad, India), which was fixed at the base of the reactor. The aeration rate was maintained at 0.5 m^3^/h. The vacuum pump, a 0.2 HP Multivac (Gurgaon, India) with a speed of 2800 RPM, was used to help the wastewater enter the ceramic membranes by suctioning of the water through the membranes. Pressure across the membranes (i.e., transmembrane pressure (TMP)) was examined using a vacuum gauge, and after 24 h of operation the membranes were subjected to chemical cleaning. The transmembrane pressure before fouling was 520 mm Hg and the TMP after fouling was 610 mm Hg. Membranes were regenerated using a wash with 10% sodium hydroxide followed by a wash with 10% sulfuric acid. After each cycle, feed and effluent waters were analyzed for turbidity, COD and DO using standard procedures. A total of three cycles were conducted for each set of membranes.

## 3. Results and Discussion

### 3.1. Membrane Characterization

**Figure 1:**
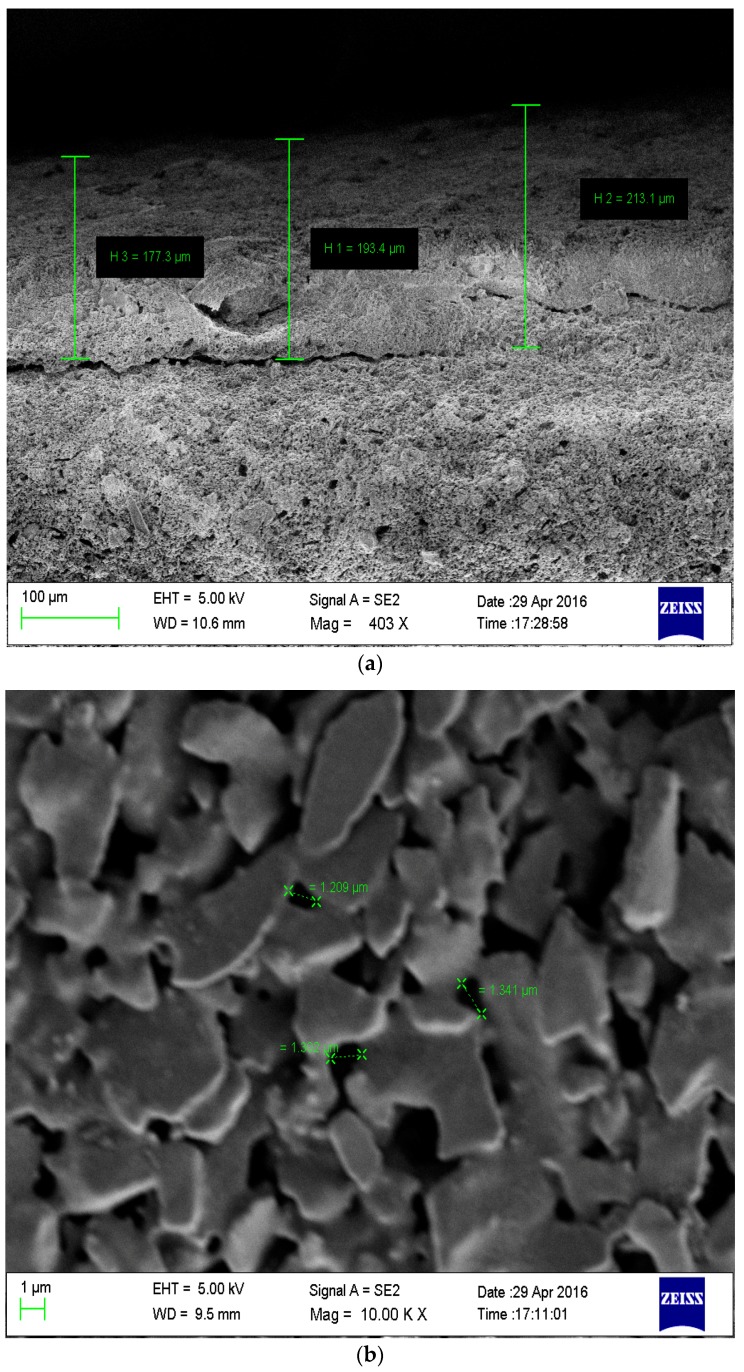

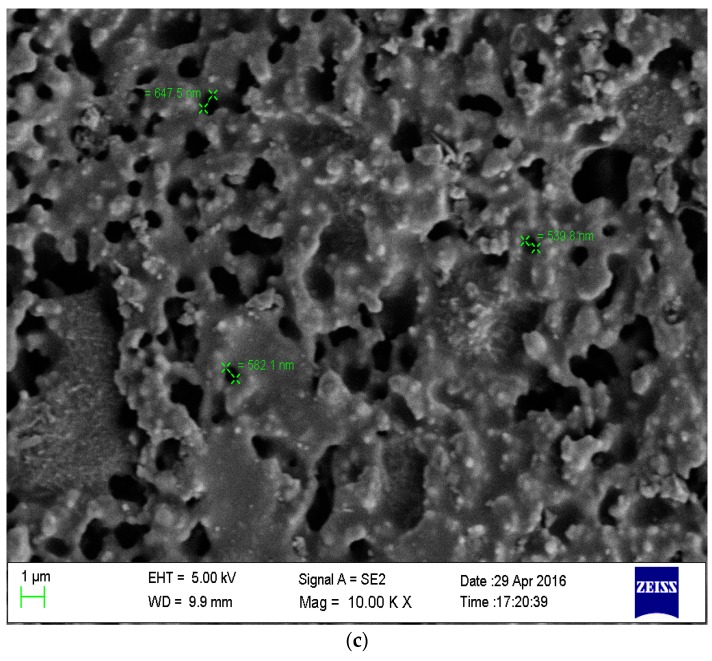
(**a**) SEM image depicting the thickness of the outer SiC coating; (**b**) SEM of uncoated membrane; (**c**) SEM of coated membrane.

Uncoated, as well as coated, membranes were characterized using scanning electron microscopy (SEM) from Tescan vega 3 (Libusina tr, Brno-Kohoutovice Czech Republic). SEM analysis of the membranes was carried out to determine the morphology, pore size and thickness of the membrane. Figure 1a represents the SEM images showing the cross section of the coated membranes in order to examine the thickness of membrane coating. The average coating thickness was found to be 200 μm. Figure 1b,c shows the magnified view at 10,000× of the uncoated and coated membranes respectively. The pores were observed on the surface of the membrane. Uniform distribution of the coating was noticed. The Figure 1b depicts that average pore size of uncoated membrane was 1.5 µm. Figure 1c shows that the average pore size for the coated membrane was 0.5 µm. Thus, the average pore size of coated membrane was less than that of the uncoated membrane.

### 3.2. Clean Water Flux Analysis

Pure water flux was determined for coated membranes. Water from the regeneration reservoir was pumped through the valve to control the pressure using the diaphragm pump and the fitting such that the water passed from outer surface to inner surface of the tubular membrane. Pressure was monitored using the pressure gauge followed by flux measurement. The procedure was repeated three times for each membrane at 1 kg·cm^−2^ and 2 kg·cm^−2^ pressure. The flux obtained for uncoated membrane at 1 kg·cm^−2^ is 1.076 × 10^3^ L·m^−2^·h^−1^ and 2.4 × 10^3^ L·m^−2^·h^−1^ for 2 kg·cm^−2^. The flux obtained for the coated membrane for 1 kg·cm^−2^ was 9.023 × 10^2^ L·m^−2^·h^−1^ and 2.168 × 10^3^ L·m^−2^·h^−1^ for 2 kg·cm^−2^. It was noticed that with the increase in pressure from 1 to 2 kg·cm^−2^, the flux value almost doubled. This implies that the flux per unit pressure remains constant. The above test was carried out for six uncoated and coated membranes, and the same trend was observed.

### 3.3. Permeate Flux Analysis

Permeate Flux refers to the volume of permeate moving through unit cross sectional area of the membrane in a unit time interval. It has dimensions (volume)(time)^−1^·(area)^−1^ and denoted by LMH (L·m^−2^·h^−1^). It was calculated using Equation (1):

(1) $$\mathrm{Permeate}\;\mathrm{Flux}=\frac{\mathrm{Volume}\;\mathrm{of}\;\mathrm{Water}\;\mathrm{Collected}}{\mathrm{Surface}\;\mathrm{Area}\;\mathrm{of}\;\mathrm{the}\;\mathrm{Membranes}\;\times \;\mathrm{Time}\;\mathrm{of}\;\mathrm{collection}}$$

**Figure 2:**
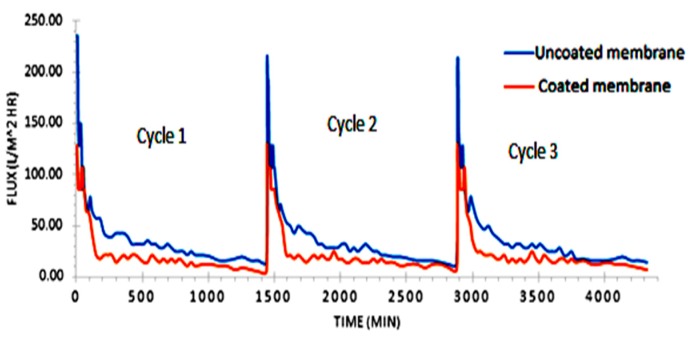
Variation of permeate flux with time of operation for both sets of membranes.

The variation of permeate flux with duration of operation (in hours) for 3 cycles of filtration was studied for both the uncoated membranes and the coated membranes. At the beginning of the first cycle of filtration, the flux was very high—235 L·m^−2^·h^−1^ for uncoated membranes and 128 L·m^−2^·h^−1^ for the coated membranes. Flux eventually dropped to 85 L·m^−2^·h^−1^ after one hour in both membranes. Thereafter, a steady value of 50 L·m^−2^·h^−1^ for uncoated membranes and 20 L·m^−2^·h^−1^ for coated membranes was observed up to 24 h. At the end of each 24-hour cycle, the membranes were regenerated using chemical wash. After regeneration, the membranes employed for the next cycle showed similar trend in flux variation with time as shown in Figure 2. The initial flux from cycle 2 was similar to that of the first cycle. Figure 2 shows the graphical comparison of flux *v*/*s* time for three cycles for both the membranes. The fouling is found to be faster for coated membranes due to smaller pore size. It can be observed that the initial flux was much higher for uncoated membranes compared to that of coated membranes. This can be attributed to the fact that the coating applied on the latter was very fine, thus reducing pore size. The smaller pore size in coated membranes makes it difficult for water to pass through the membrane encapsulated with the fouling agents.

### 3.4. Chemical Oxygen Demand (COD)

**Figure 3:**
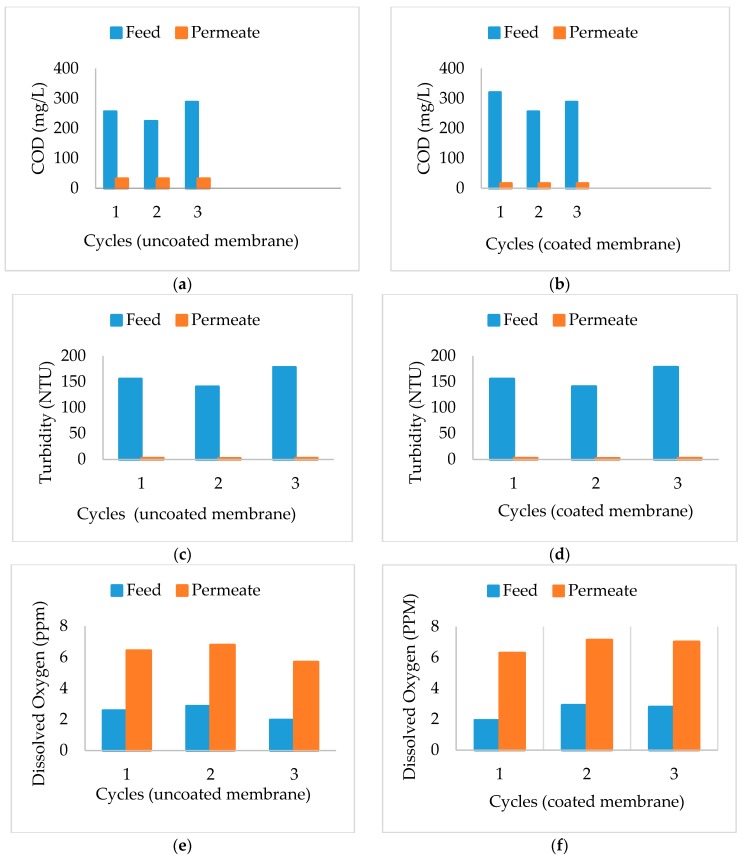
Variation in COD, Turbidity and DO for uncoated and coated membranes. (**a**) Variation in COD for uncoated membranes; (**b**) Variation in COD for coated membranes; (**c**) Variation in turbidity for uncoated membranes; (**d**) Variation in Turbidity for coated membranes; (**e**) Variation of DO for uncoated membranes; (**f**) Variation of DO for coated membranes.

Figure 3a,b shows the variation of COD for feed and permeates for the three cycles of uncoated and coated membrane respectively. COD content of the influent in the bioreactor varied from 224 to 320 mg·L^−1^. The effluent water with both coated and uncoated membranes showed a significant decrease in the COD value. For the uncoated membranes, the COD value was reduced to 32 mg·L^−1^, and for the coated membranes, the COD value was reduced to 16 mg·L^−1^, with an average reduction of about 87.33% and 94.38% for the coated and uncoated membranes, respectively. The results obtained are in good agreement with the results [46,47]. The results show that the MBR system is highly efficient in achieving reduction of COD by removing organic pollutants. The coated membranes gave a better reduction than the uncoated membranes because of their smaller pore size.

### 3.5. Turbidity

Turbidity of feed and effluent was measured using a digital nephelo-turbidity meter procured from Systronics (Ahmedabad, India), and the results are as shown in Figure 3c,d for uncoated and coated membranes, respectively. It was observed that the feed showed high values of turbidity due to the varying sizes of suspended matter and floc present in it. The clear effluent obtained from each cycle has a very low turbidity in the range 1 ppm for coated membranes and 3 ppm for uncoated. The average percentage reduction of turbidity was more than 98% for both membranes.

### 3.6. Dissolved Oxygen

Dissolved oxygen (DO) level decreases with the increase in biological oxygen demand (BOD) of the wastewater as the surplus bacteria and other microbes leading to high BOD consumes the DO in water. Dissolved oxygen availability affects the ecology of fishes and many other organisms [48,49]. Hence, DO is a notable parameter of water quality. DO concentration of about 7–11 mg/L is considered to be a very good condition for the survival of aquatic life. The dissolved oxygen in water below 5.0 mg/L would lead to a potential threat to aquatic life. The DO of the samples was analyzed using DO meter procured from Hanna Instruments (Chennai, India).

Figure 3e,f shows the DO content of the influent and effluent streams of MBR for filtration with uncoated and coated membranes. From the graph, it can be observed that the DO range for influent and effluent varied from 1.93 to 2.91 mg·L^−1^ and from 5.69 to 7.13 mg·L^−1^, respectively. The uncoated membranes revealed an average increase of 150% in DO content. The coated membrane shows an average increase of 176% in the DO content. Thus, the MBR has a capacity to produce an effluent stream with higher DO content. The DO level of the water increases for the treated water, as subjecting water to pressurized filtration results in a cavitations effect and an increase in temperature. The cavitations effect will result in the splitting of the water, the degradation of organic matter, a decrease in COD and BOD, and an increase in DO level.

### 3.7. Fouling Mechanism

Interdependency of time (*t*) and permeate volume (*V*) was analyzed for the three cycles of batch operation of MBR for both uncoated membranes and coated membranes. For the standard pore blocking model (SPBM) [50,51],

(2) tV=t[βπLρs(π8μL)12(μRmANp)12]+μRmΔPA

The above equation can be simplified as:

(3) $$\frac{t}{V}=tX_{2}+Y_{2}$$

From the above equation, it is evident that the time needed for the filtration of a unit of permeate volume is directly proportional to the product of a constant and the time period over which fluid traverses the ceramic membrane.

**Figure 4:**
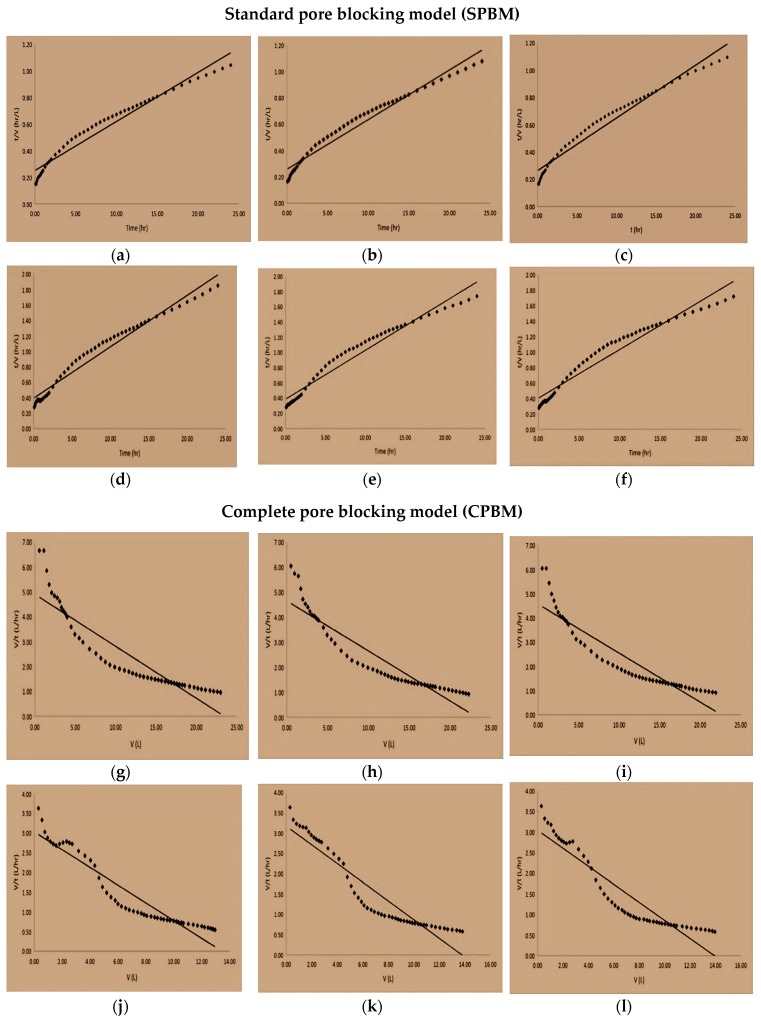
Filtration analysis of uncoated and coated membranes pursuant to the standard pore blocking model (SPBM) and the complete pore blocking model (CPBM). (**a**) Cycle 1 (uncoated membrane); (**b**) Cycle 2 (uncoated membrane); (**c**) Cycle 3 (uncoated membrane); (**d**) Cycle 1 (coated membrane); (**e**) Cycle 2 (coated membrane); (**f**) Cycle 3 (coated membrane); (**g**) Cycle 1 (uncoated membrane); (**h**) Cycle 2 (uncoated membrane); (**i**) Cycle 3 (uncoated membrane); (**j**) Cycle 1 (coated membrane); (**k**) Cycle 2 (coated membrane); (**l**) Cycle 3 (coated membrane).

The standard pore blocking model shows a linear relationship among *t* and *t*/*V*, with *X*_2_ as slope and *Y*_2_ as intercept [50]. *t/V* versus *t* values were plotted, and a linear fit was observed for uncoated membranes, as shown in Figure 4a–c, with coated membranes being as shown in Figure 4d–f. The average slope and intercept for uncoated membranes were found to be 0.0377 and 0.2573, respectively. Similarly, for the coated membranes, the average slope and intercept were found to be 0.0646 and 0.3937, respectively. The average *R*^2^ values for both were 0.9617 and 0.9564, respectively.

For the Complete Pore Plugging model (CPPM) [50,51],

(4) $$V=\frac{N_{po}}{p_{p}}\left(1-e^{-at}\right)$$

where $a=\frac{\pi \Delta P}{8\mu L}r_{p}{}^{4}p_{p}$.

From Equation (4), it is found that the CPPM model has a linear expression possessing a negative slope as a characteristic equation [50,51], which can be further simplified as

(5) $$\frac{dV}{dt}=Y_{3}-X_{3}V$$

*V/t* versus *V* values was plotted, and the data was fitted into a linear equation, as shown in Figure 4g–i. The average slope and intercept for uncoated membranes were found to be −0.2036 and 4.7056, respectively. Similarly, for the coated membranes, the average slope and intercept were found to be −0.2246 and 3.0789, respectively. The average *R*^2^ value for uncoated membranes is 0.8399 and the average *R*^2^ value for coated membranes is 0.8823. The slope, intercept and *R*^2^ value of coated and uncoated membranes with respect to SPBM are depicted in Table 1, and the slope, intercept and *R*^2^ value of coated and uncoated membranes with respect to CPPM are as shown in Table 2. By analyzing the data from Table 1 and Table 2, it is evident that the filtration data for the standard pore blocking model (SPBM) are more accurate when compared to the data for the complete pore plugging model (CPPM), due to the higher regression coefficient, *R*^2^, values. This suggests that the feed has particles whose sizes are smaller than the average pore size of the ceramic membrane. These particles enter the pore and get adsorbed onto the walls of the pore, thus reducing the effective filtration area. This results in an increase in membrane resistance. Thus, the membrane tends to lose filtration capability. The results are consistent with those reported by [50,51,52].

### 3.8. Analysis of Total Nitrogen, Total Phosphate, BOD5 and Total Suspended Solids

The influent and effluent samples were further subjected to the testing of Total Nitrogen (TN), Total Phosphate (TP), BOD5 and Total Suspended Solids (TSS). The results obtained are as follows:

#### 3.8.1. Water Effluent and Influent Quality for Uncoated Membranes

TN of the influent sample was 17 mg/L. TN of the effluent sample was 5.4 mg/L. TP of the influent sample was 4.54 mg/L and TP of the effluent sample was 1.2 mg/L. BOD5 of the influent sample was 132.63 mg/L and BOD5 of the effluent sample was 9 mg/L. TSS of the influent sample was 162.80 mg/L. TSS of the effluent sample was 6.8 mg/L.

#### 3.8.2. Water Effluent and Influent Quality for Coated Membranes

TN of the influent sample was 17 mg/L. TN of the effluent sample was 3.2 mg/L. TP of the influent sample was 4.54 mg/L and TP of the effluent sample was 0.9 mg/L. BOD5 of the influent sample was132.63 mg/L and BOD5 of the effluent sample was 5 mg/L. TSS of the influent sample was 162.80 mg/L. TSS of the effluent sample was 1.1 mg/L.

The results obtained can be effectively used in the water purification process. The coating process increases the efficacy of the treatment process. Developing nations should place importance on clean and safe environments, which are vital for the sustainable growth of the country. The present work gives a possible solution for wastewater treatment, and the usage of an eco-friendly ceramic membrane would reduce the usage of polymeric membranes, which are not cost effective and difficult to degrade after use, thus emphasizing the usage of green materials.

## 4. Conclusions

A pilot-scale setup of a submerged ceramic membrane bioreactor for microfiltration of synthetic fermentation feed was fabricated. Vacuum filtration was used instead of pressurized filtration. Aeration was found to be helpful in reducing the fouling by partially scouring the membrane surface, which resulted in the removal of biomass and organic matter from the surface of the membrane. A significant reduction in parameters like COD and turbidity of the feed water was observed. An increase in the DO content of effluent was observed. The influent feed to the MBR was black in color with high turbidity and very foul smell, whereas the effluent was colorless, clear and odorless water. A gradual decrease in permeate flux was noticed as the membrane surface became fouled due to the accumulation of suspended solids and organic matter. The cleaning of the membranes resulted in a drastic increase in permeate flux due to the regeneration of the membrane surface. The filtration data were found to fit the SPBM mathematical model more accurately than the CPPM model, which suggests that fouling was due to pore blocking of membranes by particles whose size is smaller than the pore size of the membrane. The membranes with coating had better effluent quality over membranes without coating due to the smaller pore size of the coated membrane. Future research will aim at trying different coating materials to understand the efficacy of membrane coating in wastewater treatment.

## Figures and Tables

**Table 1:** Slope, intercept and *R*^2^ values on fitting the Standard Pore Blocking model fitted for flux data.

Cycle	Uncoated Membranes			Coated Membranes		
	Slope	Intercept	*R* ^2^	Slope	Intercept	*R* ^2^
1	0.0369	0.2511	0.9574	0.0666	0.3935	0.9669
2	0.0377	0.2576	0.9637	0.0641	0.3853	0.9553
3	0.0387	0.2633	0.9641	0.063	0.4024	0.947

**Table 2:** Slope, intercept and *R*^2^ values on fitting the Complete Pore Plugging model fitted for flux data.

Cycle	Uncoated Membranes			Coated Membranes		
	Slope	Intercept	*R* ^2^	Slope	Intercept	*R* ^2^
1	−0.2088	4.8967	0.8204	−0.2251	3.0248	0.8966
2	−0.1999	4.6516	0.8543	−0.2301	3.1684	0.8825
3	−0.2021	4.5684	0.8449	−0.2187	3.0434	0.8678

## References

[1] Hosseinzadeh M., Bidhendi G.N., Torabian A., Mehrdadi N. (2013). Evaluation of membrane bioreactor for advanced treatment of industrial wastewater and reverse osmosis pretreatment. J. Environ. Health Sci. Eng..

[2] Ganesapillai M., Singh A., Simha P. (2016). Separation processes and technologies as the mainstay in chemical, biochemical, petroleum and environmental engineering: A special issue. Resour.-Effic. Technol..

[3] Ahmed Z., Cho J., Lim B.R., Song K.G., Ahn K.H. (2007). Effects of sludge retention time on membrane fouling and microbial community structure in a membrane bioreactor. J. Membr. Sci..

[4] Xing C.H., Tardieu E., Qian Y., Wen X.H. (2000). Ultrafiltration membrane bioreactor for urban wastewater reclamation. J. Membr. Sci..

[5] Gander M., Jefferson B., Judd S. (2000). Aerobic MBRs for domestic wastewater treatment: A review with cost considerations. Sep. Purif. Technol..

[6] Wang Z., Wu Z., Mai S., Yang C., Wang X., An Y., Zhou Z. (2008). Research and applications of membrane bioreactors in China: Progress and prospect. Sep. Purif. Technol..

[7] Kimura K., Hara H., Watanabe Y. (2005). Removal of pharmaceutical compounds by submerged membrane bioreactors (MBRs). Desalination.

[8] Huang X., Gui P., Qian Y. (2001). Effect of sludge retention time on microbial behavior in a submerged membrane bioreactor. Process Biochem..

[9] Shimizu Y., Okuno Y.I., Uryu K., Ohtsubo S., Watanabe A. (1996). Filtration characteristics of hollow fiber microfiltration membranes used in membrane bioreactor for domestic wastewater treatment. Water Res..

[10] Shin H.S., Kang S.T. (2003). Characteristics and fates of soluble microbial products in ceramic membrane bioreactor at various sludge retention times. Water Res..

[11] Lee J.C., Kim J.S., Kang I.J., Cho M.H., Park P.K., Lee C.H. (2001). Potential and limitations of alum or zeolite addition to improve the performance of a submerged membrane bioreactor. Water Sci. Technol..

[12] Sun D.D., Hay C.T., Khor S.L. (2006). Effects of hydraulic retention time on behavior of start-up submerged membrane bioreactor with prolonged sludge retention time. Desalination.

[13] Yamamoto K., Hiasa M., Mahmood T., Matsuo T. Direct solid-liquid separation using hollow fiber membrane in an activated sludge aeration tank. Proceedings of the Water Pollution Research and Control Brighton.

[14] Lesjean B., Rosenberger S., Schrotter J.C., Recherche A. (2004). Membrane-aided biological wastewater treatment—An overview of applied systems. Membr. Technol..

[15] Low S.L., Ong S.L., Ng H.Y. (2016). Characterization of membrane fouling in submerged ceramic membrane photobioreactors fed with effluent from membrane bioreactors. Chem. Eng. J..

[16] Wang Z., Wu Z., Yu G., Liu J., Zhou Z. (2006). Relationship between sludge characteristics and membrane flux determination in submerged membrane bioreactors. J. Membr. Sci..

[17] Rosenberger S., Krüger U., Witzig R., Manz W., Szewzyk U., Kraume M. (2002). Performance of a bioreactor with submerged membranes for aerobic treatment of municipal waste water. Water Res..

[18] Yang W., Cicek N., Ilg J. (2006). State-of-the-art of membrane bioreactors: Worldwide research and commercial applications in North America. J. Membr. Sci..

[19] Tam L.S., Tang T.W., Lau G.N., Sharma K.R., Chen G.H. (2007). A pilot study for wastewater reclamation and reuse with MBR/RO and MF/RO systems. Desalination.

[20] Meier J., Melin T. (2005). Wastewater reclamation by the PAC-NF process. Desalination.

[21] Comerton A.M., Andrews R.C., Bagley D.M. (2005). Evaluation of an MBR–RO system to produce high quality reuse water: Microbial control, DBP formation and nitrate. Water Res..

[22] Choi J.H., Ng H.Y. (2008). Effect of membrane type and material on performance of a submerged membrane bioreactor. Chemosphere.

[23] Wintgens T., Melin T., Schäfer A., Khan S., Muston M., Bixio D., Thoeye C. (2005). The role of membrane processes in municipal wastewater reclamation and reuse. Desalination.

[24] Pagana A.E., Sklari S.D., Kikkinides E.S., Zaspalis V.T. (2008). Microporous ceramic membrane technology for the removal of arsenic and chromium ions from contaminated water. Microporous Mesoporous Mater..

[25] Chiemchaisri C., Yamamoto K., Vigneswaran S. (1993). Household membrane bioreactor in domestic wastewater treatment. Water Sci. Technol..

[26] Jeong Y., Hermanowicz S.W., Park C. (2017). Treatment of food waste recycling wastewater using anaerobic ceramic membrane bioreactor for biogas production in mainstream treatment process of domestic wastewater. Water Res..

[27] Dong Z., Shang W., Dong W., Zhao L., Li M., Wang R., Sun F. (2018). Suppression of membrane fouling in the ceramic membrane bioreactor (CMBR) by minute electric field. Bioresour. Technol..

[28] Yue X., Koh Y.K.K., Ng H.Y. (2015). Effects of dissolved organic matters (DOMs) on membrane fouling in anaerobic ceramic membrane bioreactors (AnCMBRs) treating domestic wastewater. Water Res..

[29] Baker R. (2012). Microfiltration. Membrane Technology and Applications.

[30] Madaeni S.S., Fane A.G., Wiley D.E. (1999). Factors influencing critical flux in membrane filtration of activated sludge. J. Chem. Technol. Biotechnol. Int. Res. Process Environ. Clean Technol..

[31] Radjenovic J., Matosic M., Mijatovic I., Petrovic M., Barcelo D. (2008). Membrane bioreactor (MBR) as an advanced wastewater treatment technology. Emerging Contaminants from Industrial and Municipal Waste.

[32] Lee W., Kang S., Shin H. (2003). Sludge characteristics and their contribution to microfiltration in submerged membrane bioreactors. J. Membr. Sci..

[33] Howell J.A., Chua H.C., Arnot T.C. (2004). In situ manipulation of critical flux in a submerged membrane bioreactor using variable aeration rates, and effects of membrane history. J. Membr. Sci..

[34] Hai F.I., Yamamoto K., Fukushi K. (2005). Different fouling modes of submerged hollow-fiber and flat-sheet membranes induced by high strength wastewater with concurrent biofouling. Desalination.

[35] Schmidt J.E., Ahring B.K. (1996). Granular sludge formation in upflow anaerobic sludge blanket (UASB) reactors. Biotechnol. Bioeng..

[36] McCarty P.L., Bae J. (2011). Model to couple anaerobic process kinetics with biological growth equilibrium thermodynamics. Environ. Sci. Technol..

[37] Trussell R.S., Merlo R.P., Hermanowicz S.W., Jenkins D. (2006). The effect of organic loading on process performance and membrane fouling in a submerged membrane bioreactor treating municipal wastewater. Water Res..

[38] Ueda T., Hata K. (1999). Domestic wastewater treatment by a submerged membrane bioreactor with gravitational filtration. Water Res..

[39] Jin L., Ong S.L., Ng H.Y. (2013). Fouling control mechanism by suspended biofilm carriers addition in submerged ceramic membrane bioreactors. J. Membr. Sci..

[40] Chen F., Bi X., Ng H.Y. (2016). Effects of bio-carriers on membrane fouling mitigation in moving bed membrane bioreactor. J. Membr. Sci..

[41] Le-Clech P., Chen V., Fane T.A. (2006). Fouling in membrane bioreactors used in wastewater treatment. J. Membr. Sci..

[42] Jung C.W., Kang L.S. (2003). Application of combined coagulation-ultrafiltration membrane process for water treatment. Korean J. Chem. Eng..

[43] Meng F., Zhang H., Yang F., Zhang S., Li Y., Zhang X. (2006). Identification of activated sludge properties affecting membrane fouling in submerged membrane bioreactors. Sep. Purif. Technol..

[44] Zhang J., Chua H.C., Zhou J., Fane A.G. (2006). Factors affecting the membrane performance in submerged membrane bioreactors. J. Membr. Sci..

[45] Cui Z., Drioli E., Giorno L. (2014). Dip-Coating Method for Ceramic Membrane Preparation. Encyclopedia of Membranes.

[46] Liu R., Huang X., Chen L., Wen X., Qian Y. (2005). Operational performance of a submerged membrane bioreactor for reclamation of bath wastewater. Process Biochem..

[47] Rezakazemi M., Maghami M., Mohammadi T. (2018). High loaded synthetic hazardous wastewater treatment using lab-scale submerged ceramic membrane bioreactor. Period. Chem. Eng..

[48] Kramer D.L. (1987). Dissolved oxygen and fish behavior. Environ. Biol. Fishes.

[49] Gaulinger S. (2007). Coagulation Pre-Treatment for Microfiltration with. Desalination.

[50] Bolton G., LaCasse D., Kuriyel R. (2006). Combined models of membrane fouling: Development and application to microfiltration and ultrafiltration of biological fluids. J. Membr. Sci..

[51] Kumar S.M., Madhu G.M., Roy S. (2007). Fouling behaviour, regeneration options and on-line control of biomass-based power plant effluents using microporous ceramic membranes. Sep. Purif. Technol..

[52] Dufresne R., Lebrun R.E., Lavallee H.C. (1997). Comparative-study on Fluxes and performances during Paper-mill waste-water treatment with membrane bioreactor. Can. J. Chem. Eng..

